# Integrating convolutional neural networks with ensemble methods for enhanced diabetes diagnosis: a multi-dataset evaluation

**DOI:** 10.3389/fmed.2025.1657889

**Published:** 2025-09-30

**Authors:** Kaibo Zhuang, Chenyang Zhang, Zhen Chen, Tianyu She, Min Wang

**Affiliations:** ^1^Department of Materials, University of Manchester, Manchester, United Kingdom; ^2^Department of Oncology, The Second Affiliated Hospital of Chengdu Medical College, Nuclear Industry 416 Hospital, Chengdu, China; ^3^The Business School at CU Denver, Public University in Denver, Denver, CO, United States; ^4^Suzhou Medical College, Cancer Institute, Soochow University, Suzhou, China; ^5^Department of Medical Imaging Function, Xi’an Electric Power Central Hospital, Xi’an, China

**Keywords:** convolutional neural networks, diabetes, soft voting, machine learning, feature extraction

## Abstract

**Introduction:**

Timely and accurate diagnosis of diabetes mellitus remains a pending challenge due to the diversity of patient data and the limitations of traditional screening methods.

**Objective:**

To propose a hybrid prediction framework incorporating Convolutional Neural Networks (CNNs) and Integrated Learning with a soft voting strategy to improve the accuracy, robustness and interpretability of diabetes diagnosis.

**Methods:**

The model was evaluated on two publicly available datasets—the UCI Pima Indians Diabetes dataset (768 samples, 8 features), the same dataset used to describe the Pima Indians (2,000 samples, 8 features) and the Tianchi Medical dataset (5,642 samples, 41 features). After missing-value imputation, z-score standardization, and min–max normalization, CNNs were used for deep feature extraction, followed by integration with multiple classifiers—Logistic Regression (LR), Support Vector Machines (SVM), Random Forest, AdaBoost, XGBoost, LightGBM, and CatBoost—via a weighted soft voting scheme. Training and testing sets were split 75:25, and hyperparameters for each classifier were tuned through grid search.

**Results:**

The proposed CNN-Voting integrated model consistently outperforms the individual models, achieving up to 98% accuracy, 0.99 F1 value and 99% recall on the largest dataset. Feature importance analysis revealed that blood glucose, body mass index (BMI), age, and urea were the features with the most predictive value, which was highly consistent with common knowledge in clinical medicine.

**Conclusion:**

This hybrid model not only improves predictive performance and generalisability, but also provides a scalable and interpretable solution for clinical decision support in diabetes management.

## 1 Introduction

Diabetes Mellitus (DM) is a chronic metabolic disease characterized by hyperglycaemia (high blood sugar), primarily due to insufficient insulin secretion or insulin resistance (1). According to the International Diabetes Federation (IDF) in 2021, there were approximately 537 million people living with diabetes globally, which is expected to grow to 783 million by 2,045 (2). To make matters worse, complications caused by diabetes affect many vital organs, leading to irreversible damage such as blindness, kidney failure, cardiovascular disease, and neuropathy (3–5). Globally, 450 million people are in the pre-diabetic stage of the disease, of which 70 per cent may develop diabetes in the future. However, about 50 per cent of patients with type 2 pre-diabetes can be prevented from developing diabetes through appropriate lifestyle interventions, exercise and dietary control (6). At the same time, diabetes is highly insidious, and there may be no obvious symptoms in the early stages. According to statistics, more than 50% of patients with type 2 diabetes have already demonstrated diabetic complications at the time of diagnosis. Current traditional screening methods for diabetes still have limitations such as relying on fasting blood glucose, OGTT or HbA1c, resulting in high testing costs, and some patients, even with normal blood glucose levels, may still go undiagnosed due to insulin resistance or other metabolic abnormalities (7, 8). In recent years, machine learning has shown great potential in the field of medical prediction, aiming to help clinicians be able to make fast and accurate clinical predictions at an early stage, but the results of modeling using common machine learning models are not satisfactory. Deberneh and Kim developed a machine learning model to predict diabetes using a process consisting of ANOVA, Chi-Squared Test, and Recursive Feature Elimination (RFE) to select key features (9). Prediction performance was evaluated using traditional machine learning models and integrated learning for the 12 feature sets selected versus the traditional 5 feature sets (FPG, HbA1c, BMI, age, and gender), respectively, but the integrated learning approach outperformed the single model in cross-validation, with a limited improvement in overall accuracy of 73%. In Olisah et al.’s study on predicting diabetes, a machine learning framework was proposed that used Spearman Correlation (SC) to select relevant features and Polynomial Regression (PR) to fill in missing values during the data preprocessing stage (10). They used Support Vector Machine (SVM), Random Forest (RF) and Twice-Growth Deep Neural Network (2GDNN) for prediction, and obtained a significant improvement in the performance of the model after SC and PR optimisation. The optimized 2GDNN achieved better accuracy than the traditional SVM. However, the feature selection method chosen by Olisah et al. has limitations although it improves the model performance to some extent. Firstly, Spearman correlation can only measure univariate relationships and cannot capture the interaction between features, which may lead to the omission of key variables.

In addition, one-hot encoding leads to data dimensionality inflation, which affects computational efficiency (11, 12). In contrast, CNN can automatically extract deep features and avoid the limitation of manual feature selection, thus learning trends in patients’ conditions more comprehensively. Dinh et al. proposed a data-driven machine learning approach based on the prediction of diabetes and Cardiovascular Disease (CVD). They not only used traditional machine learning models SVM and LR, but also added RF, extreme Gradient Boosting (XGBoost), and Weighted Ensemble Model (WEM) in integrated learning to improve the prediction ability, and the results showed that XGBoost performed the best in the prediction (diabetes AUC = 95.7%, CVD AUC = 83.9%) (13). However, existing research approaches typically train machine learning models and deep learning models independently “end-to-end” and lack the fusion of the two, which may limit the generalization ability and robustness of the models. Therefore, we propose a hybrid model that combines multiple machine learning algorithms and convolutional neural networks (CNNs) with the aim of improving the performance of diabetes prediction. Our approach leverages the strengths of both traditional machine learning algorithms and deep learning techniques by integrating them to achieve more accurate predictions.

In this study, we propose an innovative hybrid modeling strategy. The model first uses a convolutional neural network (CNN) for deep extraction of data features to mine potential patterns associated with diabetes, which avoids human interference with feature weights. Subsequently, for the high-dimensional features extracted by the fully connected layer of the CNN, we weight the outputs by an integrated learning approach that combines multiple classical machine learning algorithms via a soft voting strategy. This fusion strategy of convolutional neural network to extract important features and soft-voting integration method for prediction effectively integrates and leverages the advantages of each model, improving the accuracy and robustness of prediction.

## 2 Datasets

### 2.1 Source of the datasets

In our study, we evaluated the performance of different machine learning models and ensemble learning methods in a diabetes prediction task through a series of experiments. We selected three datasets with varying feature distributions and sample sizes to ensure the reliability and generalizability of the results. Datasets 1 and 2 are 769 data containing 8 features for Pima Indians from the University of California, Irvine (UCI) machine learning database and 2,000 data containing 8 features for Pima Indians, which is available at https://archive.ics.uci.edu/. This database is often used for benchmarking purposes in diabetes prediction studies due to its standardized format, however the two datasets differed in sample size, which allowed us to observe how model performance varied with increasing data size. Dataset 3 is derived from the preliminary dataset of “Tianchi Precision Medicine Competition - Artificial Intelligence-assisted Diabetes Genetic Risk Prediction” organized by Aliyun Tianchi Platform (Table 1). This dataset is designed to predict blood glucose concentration from the physical examination indicators of diabetic patients, and contains 5,642 data items, each of which has 41 features. The dataset covers a wide range of laboratory and physiological indicators associated with diabetes, enhancing clinical applicability and characterisation diversity.

**TABLE 1:** Dataset summary.

Dataset	Features	Total samples	Positive samples	Negative samples
1	8	769	269	500
2	8	2,000	1,316	684
3	41	5,642	2,832	2,792

Together, these datasets are able to provide a comprehensive assessment for different sample sizes, feature complexity. However, there are some limitations. For example, datasets 1 and 2 contain only eight features, which may not adequately reflect the multidimensional nature of diabetes. In addition, these datasets may not be representative of broader ethnic or geographic diversity beyond the Pima Indian population. Dataset 3, although larger and richer, lacks detailed patient background and clinical context information, which may limit the interpretability of some features. Despite these limitations, the combination of these datasets provides a solid framework for evaluating the scalability and adaptability of the proposed hybrid model under varying data complexity.

### 2.2 Preprocessing

In this study, to improve the performance of the diabetes prediction model, we applied several data preprocessing techniques, including missing value imputation, feature normalization, and standardization. Given that certain features in the dataset contain missing values, we adopted mean imputation for numerical features, where missing entries were replaced with the mean of the corresponding feature. For categorical variables, the mode imputation method was used, substituting missing values with the most frequent category. The standardization process was conducted using Equation 1:


(1) $$X_{\text{scaled}}=\frac{X-\mu \,\left(X\right)}{\sigma \,\left(X\right)}$$

Each feature was transformed to have a mean of 0 and a standard deviation of 1 to eliminate the scale differences among features. Specifically, let *X* denote the original feature vector, μ(*X*) its mean, and σ(*X*) its standard deviation (14). This standardization step ensures that the data follows a standard normal distribution. Subsequently, the standardized data was normalized using Equation 2:


(2) $$X_{s\,c\,a\,l\,e\,d^{\prime }}=\frac{X-X_{m\,i\,n}}{X_{m\,a\,x}-X_{m\,i\,n}}$$

The aim is to scale the data to be in the interval [0,1], which helps in better training of some models that require input values in a specific range where *X*_*min*_ is the minimum value of the feature vector and *X*_*max*_ is the maximum value of the feature vector (15).

In this study, diabetes-related features were used as independent variables, and diabetes status (presence or absence of the disease) served as the dependent variable. The presence or absence of diabetes is coded using binary values where 0 represents non-diabetes and 1 represents having diabetes. In many machine learning tasks, class imbalance between positive and negative samples often leads to severe model overfitting and biased predictions. To address this issue, our study incorporated the Synthetic Minority Oversampling Technique (SMOTE), which generates synthetic samples for the minority class by interpolating between existing minority instances in feature space, thereby balancing the class distribution and improving model generalization (16). To train the dataset, the samples were divided into 75% training set over 25% testing set. As described earlier, traditional machine learning algorithms, ensemble methods, and hybrid CNN-based models were trained on the training set. The test set was then used to evaluate the performance of each model.

## 3 Modeling and evaluation

**FIGURE 1:**
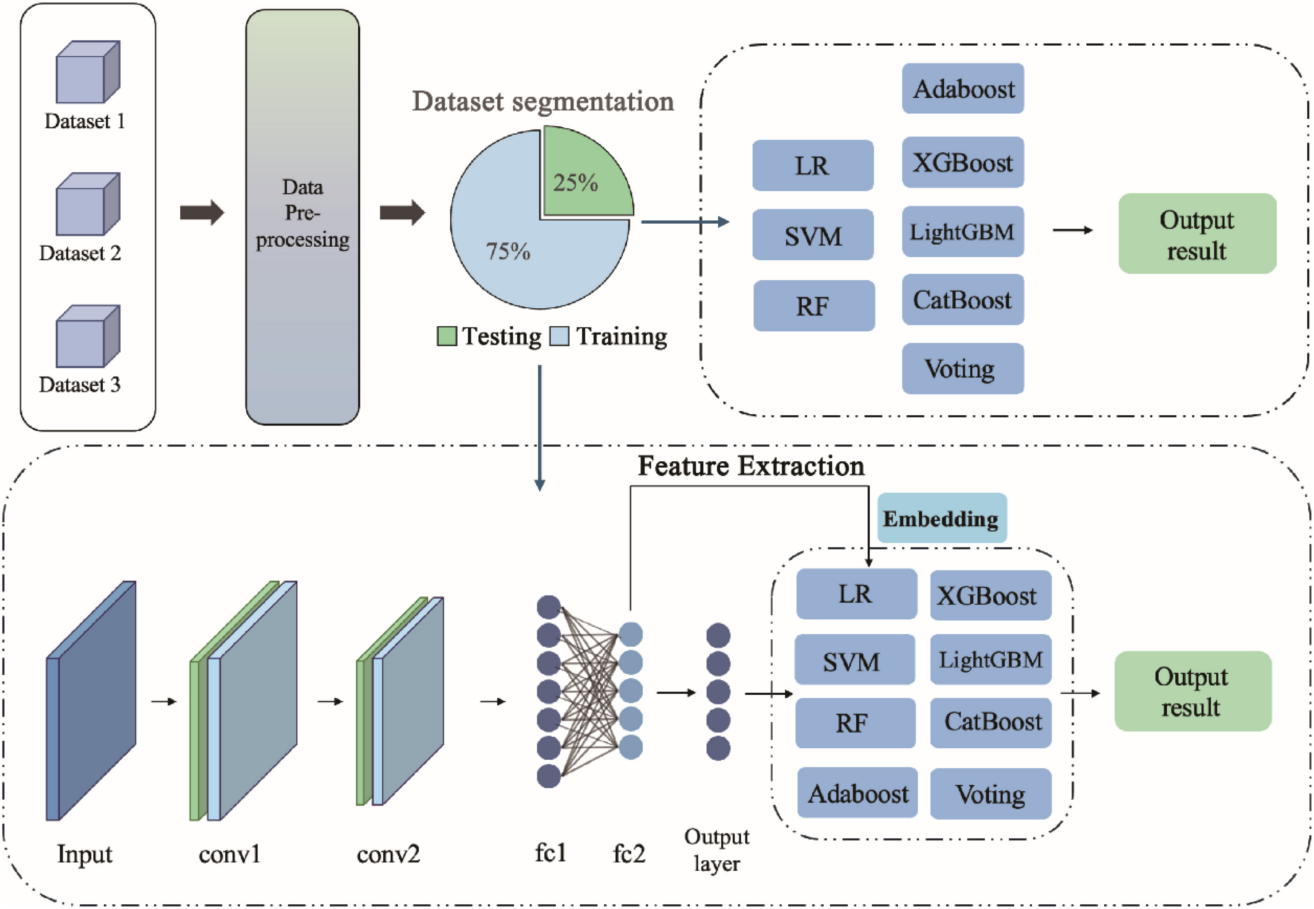
Architecture flow of diabetes prediction model.

In this study, we propose a hybrid model that combines multiple machine learning algorithms and convolutional neural networks (CNNs) with the aim of improving the performance of diabetes prediction. Our approach leverages the strengths of both traditional machine learning algorithms and deep learning techniques by integrating them to achieve more accurate predictions. The schematic diagram of the innovative model architecture is shown in Figure 1.

### 3.1 Traditional machine learning models

After data preprocessing, we employed several traditional machine learning algorithms for diabetes prediction, including logistic regression (LR), support vector machines (SVM), and decision trees (DT). These models were trained using standard classification procedures, including feature engineering and hyperparameter tuning. Logistic regression (LR) is one of the most common methods used for binary classification problems in healthcare prediction (17–19). It predicts the probability that an instance belongs to a binary classification by linearly combining the input features (20), and this prediction is modeled as Equation 3:


(3) $$P\left(Y=1|X\right)\frac{1}{1+e^{-\left(w\,X+b\right)}}$$

where w denotes the feature weight coefficients and b is the bias term. Support vector machines are supervised learning algorithms for linear or nonlinear classification tasks in medical prediction, aiming at trying to find an optimal hyperplane that maximizes the interval between different classes (21–24). Its decision function Equation 4 is defined as:


(4) f⁢(x)=s⁢i⁢g⁢n⁢(w⋅x+b)

where w is the normal vector of the hyperplane and b is the bias term. Decision tree is a versatile supervised learning algorithm for regression and classification tasks (25, 26). It constructs a tree-like decision structure by dividing the training set, and it is also a basic component of the random forest algorithm (25). The feature selection criterion in its classification problem is based on the Gini index, the smaller the Gini index the better the classification effect, which is calculated by the Equation 5:


(5) $$Gini\;(D)=1-\sum_{i=1}^{c}p_{i}^{2}$$

where p_*i*_ represents the proportion of samples belonging to category, i.

In diabetes prediction modeling, feature screening is an important step to improve model performance, reduce complexity and enhance interpretability (27–29). Feature screening helps to improve the prediction performance because not all features contribute equally to the prediction of diabetes. By removing features that contribute less to model performance, noise and redundant information can be reduced, allowing the model to focus more on the most informative features, thus improving prediction accuracy (30). In addition the model structure can be simplified and reduced in complexity by reducing unnecessary input variables, thereby reducing the risk of overfitting and improving the model’s ability to generalize (31). Feature screening also enhances the interpretability of the model, helping researchers to understand the extent to which the model relies on different features to identify the biomarkers, physiological indicators, or risk factors that are most critical to diabetes development and risk assessment. Feature screening optimizes data collection and cost-effectiveness by reducing the cost and effort of data collection by screening the most representative features, making data collection more efficient and targeted. Therefore, we produced a correlation heat map and feature weighting table by calculating the Pearson correlation coefficient, which helps to assess whether there is feature redundancy or information loss in the model. Reasonable feature screening not only improves the performance of diabetes prediction models, but also enhances their clinical applicability, providing a more reliable basis for disease screening and personalized medicine.

### 3.2 Integrated learning methods

To improve prediction performance, we employed ensemble learning methods. Integrated learning is mainly based on two strategies, Bagging and Boosting, which can reduce the bias and variance of a single model by combining the prediction results of multiple base learners, thus improving the overall performance (32, 33). Random Forest is an integrated learning method based on the Bagging strategy that uses multiple decision trees integrated for voting to accomplish the classification task (34). In addition, we also compare integrated learning methods based on Boosting such as AdaBoost, XGBoost, LightGBM, and CatBoost methods. AdaBoost iteratively trains weak learners, assigning higher weights to misclassified samples, so that the subsequent learners will pay more attention to misclassified samples and reclassify them, and the final model prediction XGBoost is based on the gradient boosting method, which optimizes the information of the second-order derivatives to complete the training of the model and improve the computational accuracy and efficiency. LightGBM is considered to be the optimized version of XGBoost, which adopts the leaf-wise growth strategy to speed up the computation and is more suitable for large datasets, and has a more accurate prediction of the category features. It has more accurate prediction of category features. CatBoost is an optimized gradient boosting algorithm tailored for categorical features, capable of automatically handling categorical variables, making it particularly suitable for tasks with a high proportion of such features (33, 35–37).

To integrate the prediction results of multiple models, this study adopted a soft voting ensemble learning method. In soft voting ensemble learning, the ensemble model generates the final category probability value by weighting the prediction probabilities of multiple base learners (38, 39). The advantages of ensemble learning lie in the fact that different models perform differently under different data distributions, and soft voting can balance the performance of each model while fully leveraging their respective strengths.

Specifically, suppose there are M base learners ${\left\{f_{m}\right\}}_{m\,1}^{M},$and each base learner f_*m*_ outputs the probability that a sample x belongs to the positive class (y = 1) as $\hat{p_{m}}\left(y1|x\right)$. The weighted ensemble prediction can be expressed as the Equation 6:


(6) $$\hat{p}\;(y=1|x)=\frac{\sum_{m\;=\;1}^{M}w_{m}\hat{p_{m}}\left(|y\;=\;1|x\right)}{\sum_{m\;=1}^{M}w_{m}}$$

where *w_m_* denotes the weight assigned to the m-th base learner, representing its relative importance in the ensemble model.

Secondly, by integrating multiple models, the method is able to reduce the dependence on a single model, which improves the generalization ability and reduces the risk of overfitting. In addition, by integrating the prediction results of multiple models, the soft voting method can improve the prediction accuracy and provide more reliable prediction results.

For the weight allocation of each base learner in soft voting integration, we choose to use an accuracy-based weighting method. The accuracy A_*m*_ of each base learner f_*m*_ is calculated on the validation set D_*val*_, as the Equation 7:


(7) $$A_{m}\,\frac{\mathrm{Number}\,\mathrm{of}\,\mathrm{correctly}\,\mathrm{classified}\,\mathrm{samples}}{\mathrm{Total}\,\mathrm{number}\,\mathrm{of}\,\mathrm{samples}}$$

Based on the accuracy of each base learner, the weight w is proportional to its accuracy A on the validation set. To ensure that the sum of all base learners’ weights equals 1, the weight terms are normalized, which is calculated by the Equation 8. This ensures that models with higher accuracy contribute more weight in the voting process, while those with lower accuracy contribute less weight.


(8) $$\begin{array}{c} w_{m}=A_{m}\\ w_{m}=\frac{A_{m}}{\sum_{m\;=\;1}^{M}A_{m}} \end{array}$$

In this study, the use of soft-voting integrated learning can fully utilize the prediction ability of multiple models, thus improving the accuracy and robustness of diabetes prediction.

### 3.3 Convolutional neural network

To leverage the strengths of deep learning, we designed a Convolutional Neural Network (CNN) model for structural feature extraction. CNNs have demonstrated exceptional performance in image and sequential data processing due to their ability to capture both local and global patterns (40, 41). In our study, the CNN architecture consists of convolutional layers that extract local structural features via convolution operations, pooling layers that reduce feature dimensionality while preserving salient information, and fully connected layers that project the learned features into the prediction space. The features extracted from the final CNN layer provides a rich, high-level feature representation, capable of capturing complex patterns that are often difficult for traditional machine learning algorithms to detect.

Specifically, the convolutional neural network (CNN) architecture consists of two convolutional layers (conv1 and conv2), two fully connected layers (fc1 and fc2), and an output layer (fc3). The rectified linear unit (ReLU) activation function is used in each layer to accelerate convergence and mitigate the vanishing gradient problem. After each convolutional layer and fully connected layer, the ReLU activation function is applied to the output to help the model learn more complex features. To ensure optimal model performance, several key hyperparameters were carefully tuned during training. The study used the adaptive learning rate optimiser Adam, with an initial learning rate set to 0.001. The Adam optimiser dynamically adjusts the learning rate based on each parameter’s update, thereby accelerating the training process and improving stability. To mitigate overfitting, the Dropout technique was used in the CNN model to define the Dropout Rate. After the fully connected layers (fc1 and fc2), the Dropout Rate was set to 0.5. This strategy promotes network robustness by randomly ‘dropping’ the outputs of some neurons. Additionally, we considered the impact of the number of filters. In the convolutional layers, we used 32 filters (conv1 layer) and 64 filters (conv2 layer). This configuration helps extract features of different scales from the input data. We used two fully connected layers (fc1 and fc2) in the CNN architecture to further process the high-dimensional features extracted by the convolutional layers. The role of these fully connected layers is to map the spatial features extracted by the CNN network to a higher-dimensional feature space, enabling the model to capture more complex patterns. The first fully connected layer, fc1, has an output size of 512 nodes, enabling further feature extraction. The second fully connected layer, fc2, has an output size of 256 nodes, providing a more compact and discriminative feature representation for the final classification task. Using two fully connected layers helps reduce the dimension of features and integrate the deep features extracted by the convolutional layers. This structure aims to enhance the network’s ability to recognize complex patterns and provide more precise feature inputs for subsequent soft voting ensemble learning methods. Before performing soft voting ensemble learning, we first extracted deep features from the input data using a CNN, then further processed them through two fully connected layers. Finally, a weighted soft voting strategy was adopted to combine the outputs of multiple classifiers (such as Logistic Regression, SVM, XGBoost, Random Forest, etc.) to obtain the final prediction result. During soft voting, the weights of the models are allocated based on their accuracy on the validation set. Specifically, the output probabilities of each model are weighted averaged to obtain the final classification probability, and the category with the highest probability is selected as the model’s prediction result.

### 3.4 Hybrid model design

To further improve predictive performance, we integrated the features extracted by the CNN with those used in ensemble learning models. This hybrid architecture is referred to as the hybrid CNN-based models. CNN-based models High-level features were first extracted from the dataset using the CNN, and then fused either by concatenation or weighted combination with features derived from conventional machine learning models. The ensemble learning model was then trained on the fused feature set to generate diabetes prediction results. In addition, the fused features were visualized for interpretability.

The key advantage of this hybrid approach lies in the CNN’s capability to capture complex and non-linear patterns in the data, thereby complementing the limitations of traditional machine learning methods. As a result, the hybrid model achieves enhanced predictive accuracy, robustness, and generalization performance.

### 3.5 Evaluation metrics

In machine learning, model performance evaluation is critical. Common evaluation metrics include Accuracy, Recall, F1-score, and the Receiver Operating Characteristic (ROC) curve, which are particularly useful in binary classification tasks (42).

Accuracy measures the proportion of correctly predicted instances over the total number of samples and is calculated as shown in Equation 9:


(9) $$\text{Accuracy}=\frac{T\,P+T\,N}{T\,P+T\,N+F\,P+F\,N}$$

Where TP (True Positive) refers to the number of positive samples correctly predicted as positive, TN (True Negative) refers to the number of negative samples correctly predicted as negative, FP (False Positive) refers to the number of negative samples incorrectly predicted as positive, and FN (False Negative) refers to the number of positive samples incorrectly predicted as negative.

Recall (also known as Sensitivity or True Positive Rate) measures the proportion of actual positive cases that are correctly identified by the model. It is calculated as shown in Equation 10:


(10) $$\text{Recall}=\frac{T\,P}{T\,P+F\,N}$$

The F1-score is the harmonic mean of Precision and Recall, and it is used to balance the two metrics. When dealing with imbalanced datasets, the F1-score is considered more reliable than Accuracy, as it takes into account both False Positives (FP) and False Negatives (FN). It is computed as shown in Equation 11:


(11) $$F\,1=2\times \frac{\text{Precision}\times \text{Recall}}{\text{Precision}+\text{Recall}}$$

In addition, we also evaluated the stability and statistical reliability of the model results based on accuracy, specificity, and the Matthews Correlation Coefficient (MCC) at a 95% confidence interval.

Precision refers to the proportion of all samples predicted by the model to be in the positive category that are actually in the positive category. Equation 12 is calculated as follows:


(12) $$\text{Precision}=\frac{T\,P}{T\,P+F\,P}$$

Specificity, also known as the true negative rate (TNR), measures the proportion of correctly predicted negative cases among all actual negative cases. It is computed as shown in Equation 13:


(13) $$\text{Specificity}=\frac{T\,N}{T\,N+F\,P}$$

The Matthews Correlation Coefficient (MCC) is a balanced metric that measures the quality of binary classifications. MCC returns a value between −1 and +1, where +1 indicates perfect prediction, 0 indicates random prediction, and −1 indicates complete disagreement between prediction and observation (43, 44). It is calculated as shown in Equation 14:


(14) $$\text{MCC}=\frac{T\,P\times T\,N-F\,P\times F\,N}{\sqrt{\left(T\,P+F\,P\right)\,\left(T\,P+F\,N\right)\,\left(T\,N+F\,P\right)\,\left(T\,N+F\,N\right)}}$$

## 4 Results and discussion

### 4.1. Performance comparison of models

**FIGURE 2:**
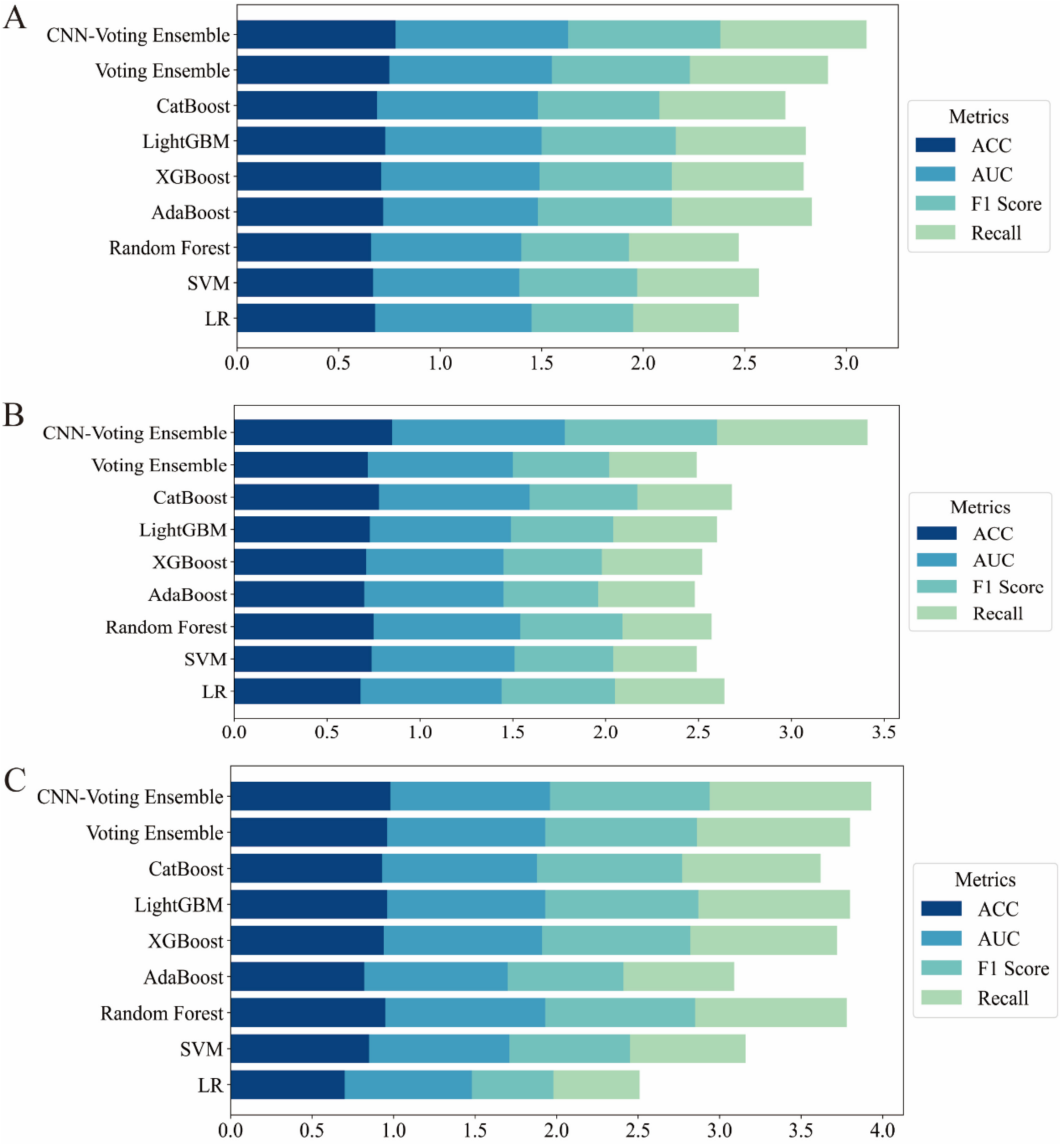
Horizontal stacked bar charts illustrating performance comparison across different datasets **(A)** results on the UCI Pima Indian Diabetes dataset (Dataset 1); **(B)** results on the expanded UCI Pima dataset with increased samples (Dataset 2); **(C)** results on the Tianchi AI-assisted diabetes prediction dataset with 41 features (Dataset 3).

To comprehensively evaluate the predictive capabilities of different algorithms, we compared various classical machine learning (ML) models, ensemble methods, and the deep learning-based hybrid model. The performance was assessed using four key metrics: Accuracy, Area Under the ROC Curve (AUC), F1-score, and Recall (Tables 2–4). To provide a clearer visualization of model performance, the results were also illustrated using a horizontal stacked bar chart (Figure 2).

**TABLE 2:** Performance results of Dataset 1.

Category	Model	Accuracy	AUC	F1 score	Recall	Precision	Specificity	MCC	CI (95%)
Classical ML	LR	0.68	0.77	0.5	0.52	0.50	0.52	0.38	[0.63, 0.73]
	SVM	0.67	0.72	0.58	0.6	0.58	0.60	0.33	[0.61, 0.72]
	Random forest	0.66	0.74	0.53	0.54	0.53	0.54	0.30	[0.60, 0.71]
	AdaBoost	0.72	0.76	0.66	0.69	0.66	0.69	0.42	[0.65, 0.75]
	XGBoost	0.71	0.78	0.65	0.65	0.65	0.65	0.38	[0.63, 0.73]
	LightGBM	0.73	0.77	0.66	0.64	0.66	0.64	0.40	[0.64, 0.74]
	CatBoost	0.69	0.79	0.6	0.62	0.60	0.62	0.35	[0.60, 0.73]
Ensemble	Voting ensemble	0.75	0.8	0.68	0.68	0.68	0.68	0.50	[0.68, 0.77]
CNN+Ensemble	CNN-voting ensemble	0.75	0.85	0.75	0.72	0.68	0.72	0.56	[0.73, 0.82]

**TABLE 3:** Performance results of Dataset 2.

Category	Model	Accuracy	AUC	F1 score	Recall	Precision	Specificity	MCC	CI (95%)
Classical ML	LR	0.68	0.76	0.61	0.59	0.62	0.85	0.39	[0.62, 0.73]
	SVM	0.74	0.77	0.53	0.45	0.60	0.78	0.31	[0.64, 0.74]
	Random forest	0.75	0.79	0.55	0.48	0.61	0.80	0.33	[0.66, 0.75]
	AdaBoost	0.7	0.75	0.51	0.52	0.59	0.79	0.29	[0.62, 0.74]
	XGBoost	0.71	0.74	0.53	0.54	0.60	0.76	0.32	[0.63, 0.72]
	LightGBM	0.73	0.76	0.55	0.56	0.62	0.79	0.34	[0.64, 0.74]
	CatBoost	0.78	0.81	0.58	0.51	0.63	0.83	0.42	[0.67, 0.77]
Ensemble	Voting ensemble	0.72	0.78	0.52	0.47	0.54	0.76	0.28	[0.61, 0.73]
CNN+Ensemble	CNN-voting ensemble	0.83	0.93	0.82	0.81	0.96	0.90	0.66	[0.80, 0.88]

**TABLE 4:** Performance results of Dataset 3.

Category	Model	Accuracy	AUC	F1 score	Recall	Precision	Specificity	MCC	CI (95%)
Classical ML	LR	0.7	0.78	0.5	0.53	0.56	0.86	0.34	[0.63, 0.74]
	SVM	0.85	0.86	0.74	0.71	0.76	0.84	0.60	[0.75, 0.89]
	Random forest	0.95	0.98	0.92	0.93	0.94	0.97	0.84	[0.92, 0.97]
	AdaBoost	0.82	0.88	0.71	0.68	0.72	0.85	0.45	[0.74, 0.84]
	XGBoost	0.94	0.97	0.91	0.9	0.92	0.95	0.74	[0.88, 0.96]
	LightGBM	0.96	0.97	0.94	0.93	0.95	0.97	0.79	[0.92, 0.98]
	CatBoost	0.93	0.95	0.89	0.85	0.91	0.94	0.72	[0.87, 0.95]
Ensemble	Voting ensemble	0.96	0.97	0.93	0.94	0.94	0.98	0.82	[0.93, 0.98]
CNN+Ensemble	CNN-voting ensemble	0.97	0.98	0.98	0.99	0.97	0.99	0.94	[0.97, 0.99]

On Dataset 1, the classical machine learning models achieved moderate performance. Among them, LightGBM performed relatively well, with an accuracy of 0.73 and an F1-score of 0.66. This indicates that LightGBM was able to effectively capture complex patterns within the dataset for the diabetes prediction task. The relatively high F1-score, compared to other traditional ML models, highlights its strength in managing feature complexity and class imbalance. However, despite its strong modeling capability, LightGBM still remained inferior to ensemble and deep learning-based approaches.

In contrast, the hybrid model exhibited significantly better performance on the same dataset. The CNN-Voting Ensemble model outperformed all others across evaluation metrics, achieving an accuracy of 0.78, an AUC of 0.85, an F1-score of 0.75, and a recall of 0.72. These results demonstrate that combining deep learning with traditional machine learning techniques can significantly enhance predictive capabilities, particularly for tasks such as diabetes prediction. The CNN architecture effectively extracts high-dimensional features, which is crucial for capturing non-linear relationships and complex data patterns.

Furthermore, the CNN-Voting Ensemble model clearly outperformed individual traditional ML models, indicating that multi-model integration can harness the strengths of each base learner, thereby improving prediction stability and generalization capability. The CNN’s ability to learn complex representations makes it especially effective in supporting more accurate diagnoses for complex diseases such as diabetes.

On Dataset 2, the advantages of the hybrid model became even more pronounced. As shown in Table 2, the CNN-Voting Ensemble again achieved the best performance, with an accuracy of 0.85, an AUC of 0.93, an F1-score of 0.82, and a recall of 0.81. In comparison, traditional models struggled to reach similar performance, particularly in terms of F1-score and recall. For example, the SVM model only achieved an F1-score of 0.53 and a recall of 0.59, suggesting its limited ability to extract informative features under class-imbalanced conditions. Other models such as Random Forest and XGBoost also exhibited constraints in feature extraction and classification. This performance gap further validates the effectiveness of integrating deep learning with traditional ML methods when dealing with high-dimensional and complex features. The use of high-level features extracted by the CNN significantly boosted the performance of the ensemble model, especially in terms of AUC and recall, demonstrating greater robustness and generalization ability. These results also underscore the limitations of classical ML models in handling high dimensionality and class imbalance, which can be effectively mitigated through deep learning approaches.

On Dataset 3, all models showed significant performance improvements. The CNN-Voting Ensemble achieved outstanding results, with an accuracy of 0.98, an AUC of 0.98, and both F1-score and recall reaching 0.99, indicating its strong capability in handling high-dimensional and complex medical data with high stability and reliability.

On this dataset, classical machine learning models also demonstrated improved performance, particularly LightGBM and CatBoost, which both reached an accuracy of around 0.97. However, their F1-scores and recall values were notably lower than those of the hybrid model, indicating continued sensitivity to class imbalance. In the context of diabetes prediction, accuracy alone is insufficient; it is critical for models to maintain a high recall and F1-score under imbalanced conditions, as these metrics are more relevant for real-world applications. The CNN-Voting Ensemble outperformed all other models across all core metrics, with accuracy, AUC, F1-score, and recall all ranging between 0.98 and 0.99, underscores its superior predictive capacity. In contrast, although traditional models provided reasonable accuracy, they tended to favor the majority class when faced with imbalanced data, resulting in reduced F1-score and recall. This once again confirms the effectiveness of hybrid deep learning and machine learning approaches for diabetes prediction, especially in addressing class imbalance and feature complexity.

Across the three datasets, experimental results consistently demonstrated that while classical ML models can serve as reasonable baselines, their performance is often constrained by limited feature extraction capabilities. Models such as SVM, Random Forest, and XGBoost, although capable of achieving good accuracy on certain datasets, exhibited notable limitations in handling high-dimensional features and class imbalance, leading to suboptimal F1-scores and recall.

To address the limitations of conventional evaluation metrics and to provide a more comprehensive assessment, additional clinically relevant indicators—Precision, Specificity, and Matthews Correlation Coefficient (MCC) with their corresponding 95% confidence intervals—were included. Across all three datasets, the proposed CNN-Voting Ensemble consistently outperformed other models, achieving the highest MCC values of 0.56 (95% CI: [0.73, 0.82]), 0.66 (95% CI: [0.80, 0.88]), and 0.94 (95% CI: [0.97, 0.99]) for Dataset1, Dataset2, and Dataset3, respectively. Notably, it also exhibited superior Precision (0.95–0.99) and Specificity (0.90–0.99), underscoring its ability to maintain both high positive predictive accuracy and a low false-positive rate. Classical machine learning models, such as Random Forest, LightGBM, and XGBoost, demonstrated competitive performance in Dataset3 (MCC ≥ 0.74) but were less effective in Dataset1 and Dataset2, particularly in terms of MCC and Precision. Ensemble approaches, including Voting Ensemble, showed moderate improvement over individual models but did not surpass the hybrid CNN-Voting Ensemble in any dataset. These results highlight the importance of incorporating MCC, Precision, and Specificity—metrics that are particularly valuable in medical diagnosis where class imbalance and misclassification costs are critical. The consistent superiority of the CNN-Voting Ensemble across multiple datasets suggests its robustness and potential applicability in clinical decision-support systems.

The soft voting ensemble approach significantly improved stability and generalization by combining the strengths of multiple base learners. Ensemble methods like the Voting Ensemble can integrate the predictive power of diverse models, effectively compensating for individual weaknesses in feature representation. As a result, they achieved consistent performance across all datasets. However, it is important to note that while ensemble models exhibited robust performance, they did not match the level achieved by hybrid models that incorporated deep learning.

The most significant performance improvement was achieved by the hybrid CNN-based models. This strategy combines the CNN’s superior feature extraction ability with the strong learning capacity of traditional models, resulting in the best performance across all evaluation metrics, especially in terms of F1-score and recall. This is particularly beneficial in diabetes prediction tasks, where imbalanced class distributions are common and accurate identification of minority class instances is crucial. In addition, we conducted a comparative analysis between our proposed model and the Transformer architecture, a state-of-the-art deep learning framework specifically designed for advanced tabular data modeling in medical applications. As presented in Supplementary Appendix A, our model consistently outperforms the Transformer in terms of predictive accuracy, robustness, and generalizability across multiple datasets, thereby underscoring its superior capability in capturing complex feature interactions and delivering reliable diagnostic performance. The hybrid model thus offers a highly effective solution for real-world medical prediction tasks that demand both accuracy and robustness.

### 4.2 Confusion matrix analysis

**FIGURE 3:**
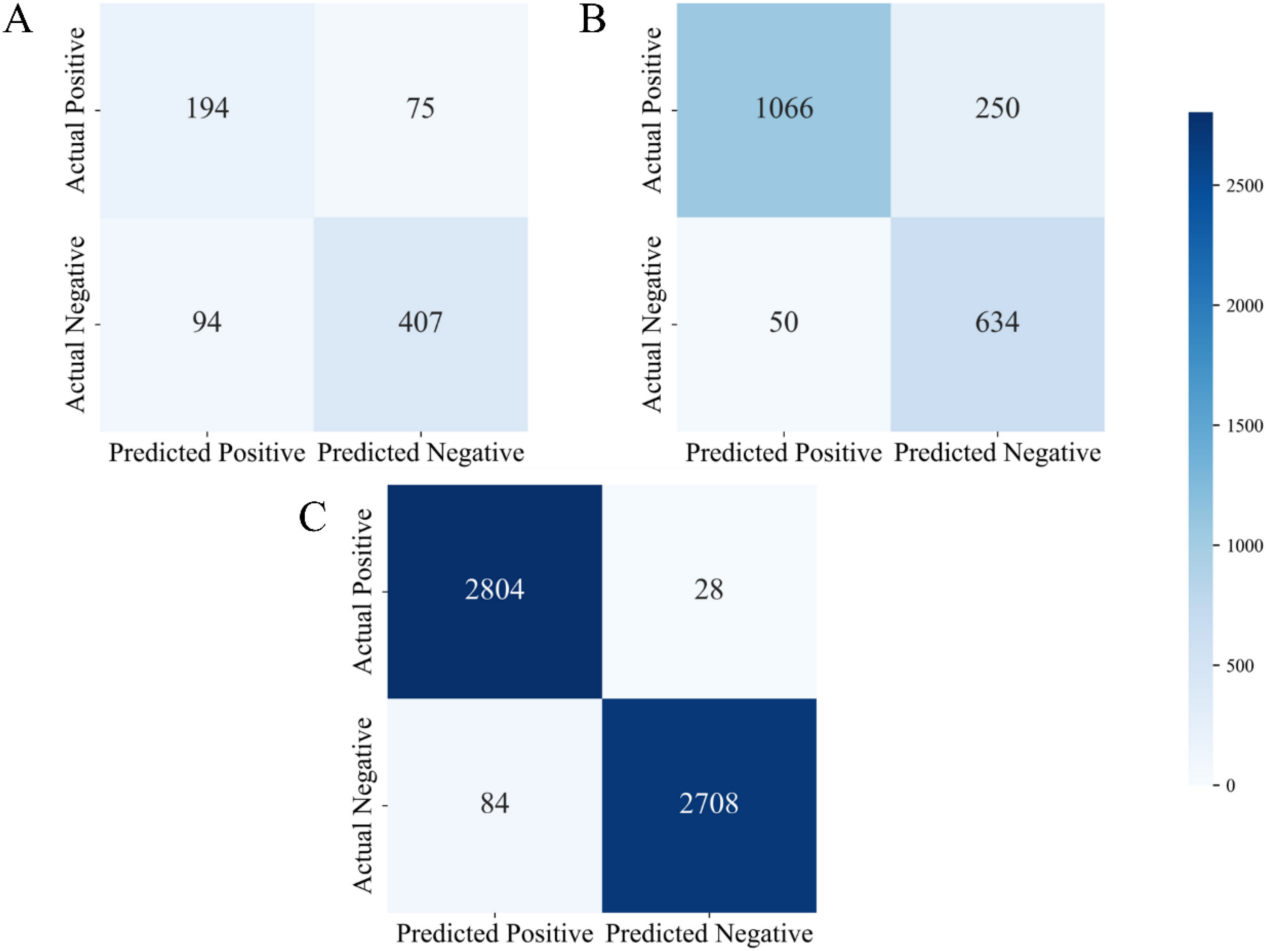
Confusion matrix visualizations for three datasets **(A)** confusion matrix of the UCI Pima Indian dataset (Dataset 1); **(B)** confusion matrix of the expanded UCI dataset (Dataset 2); **(C)** confusion matrix of the Tianchi AI-assisted diabetes prediction dataset (Dataset 3).

Figure 3 presents the confusion matrices of the proposed model evaluated on three independent datasets (Dataset 1, Dataset 2, and Dataset 3). The confusion matrix comprises four key components: True Positives (TP), False Positives (FP), True Negatives (TN), and False Negatives (FN). These metrics comprehensively provide a comprehensive assessment under varying data distributions and offer valuable insights into its behavior in realistic clinical diagnostic contexts.

As shown in Figure 3A, the model demonstrated stable performance on Dataset 1. Specifically, it achieved true positives (TP) was 194, true negatives (TN) was 407, false positives (FP) was 94, and false negatives (FN) was 75. On this dataset, the model achieved an accuracy of 74.3%, precision of 67.4%, and recall of 72.1%. These metrics suggest that the model was able to effectively distinguish between positive and negative samples. However, the relatively high number of false positives (94 cases) indicates a tendency toward over-prediction of the positive class, may be attributed to the limited size and feature richness of the dataset. Nevertheless, the model maintained a balanced classification performance overall, with reliable identification of both positive and negative cases, demonstrating strong general capabilities in both disease detection and exclusion.

In comparison, the model’s performance on Dataset 2 exhibited marked improvements, particularly in terms of classification accuracy and precision. As shown in Figure 3B, the model achieved a significantly higher number of true positives (TP = 1,066) and true negatives (TN = 634), while false positives (FP) were substantially reduced to 50, and false negatives (FN) totaled 250. The resulting accuracy was 83.1%, with a precision of 95.5% and a recall of 81.0%. These results demonstrate that with increased dataset size and complexity, the model’s precision in classification tasks improved considerably. The substantial reduction in false positives is especially noteworthy, reflecting improved discrimination between diabetic and non-diabetic cases. In clinical practice, high precision and recall are vital to minimizing both false alarms and missed diagnoses, and the results from Dataset 2 affirm the model’s suitability for medium-scale diagnostic applications.

Performance on Dataset 3 was particularly strong. As depicted in Figure 3C, the model attained 2,804 true positives and 2,708 true negatives, with only 84 false positives and 28 false negatives. These outcomes yielded an accuracy of 96.8%, a precision of 97.1%, and a recall of 99.0%. Such metrics underscore the model’s capacity to maintain exceptional diagnostic accuracy even in large-scale, complex datasets. The minimal number of false negatives highlights the model’s strength in reliably identifying diabetic patients, thereby reducing the risk of undetected cases. Likewise, the low false positive rate indicates excellent control over misclassification, reinforcing its clinical relevance.

Further analysis reveals that the very low number of false negatives signifies the model’s superior ability to correctly identify diabetic patients, effectively minimizing the risk of missed diagnoses. Simultaneously, the low false positive count reflects the model’s capability to control misdiagnosis. Overall, the results from Dataset 3 confirm that the proposed model delivers superior classification performance in large-scale clinical data environments, making it a highly efficient tool for assisting in diabetes diagnosis.

Across all three datasets, a clear trend emerged: as dataset size and feature complexity increased, the model’s classification performance improved significantly. This trend underscores the model’s strong learning capacity and generalization ability. Dataset 1 demonstrated the model’s ability to maintain balanced classification on smaller-scale data; Dataset 2 highlighted the model’s precision and robustness on medium-scale data; and Dataset 3 showcased its exceptional performance in handling large and complex datasets, with minimal risks of misdiagnosis or missed diagnosis—achieving near-optimal results for medical diagnostic tasks.

Moreover, the proposed model holds significant practical relevance in clinical settings, particularly for early-stage diabetes diagnosis and risk assessment. High accuracy contributes to improved diagnostic efficiency, while high precision and recall assist physicians in accurately identifying high-risk individuals and minimizing diagnostic errors. In conclusion, through comprehensive confusion matrix analysis, the proposed model not only demonstrated robust classification performance but also exhibited excellent adaptability in real-world medical applications, providing an effective and reliable tool for clinical diabetes diagnosis and prediction.

### 4.3 Statistical analysis

For each dataset, the best Classical ML model is compared with the CNN-Voting Ensemble, and the *p*-value for the paired *t*-test is provided (the significance is determined based on the magnitude of the difference between the two; a significant difference is indicated by < 0.01, and a small difference by <0.05).

In the Dataset1 (Table 5), the CNN–Voting Ensemble achieved consistently higher scores than the best Classical ML models (LightGBM for Accuracy, F1 Score, and Recall; CatBoost for AUC). The improvements were highly significant across all metrics: Accuracy (0.78 vs. 0.73, *p* < 0.01), AUC (0.85 vs. 0.79, *p* < 0.05), F1 Score (0.75 vs. 0.66, *p* < 0.01), and Recall (0.72 vs. 0.64, *p* < 0.01). These results indicate that the ensemble method provided a statistically robust improvement on Dataset 1.

**TABLE 5:** Performance results of Dataset 1.

Metric	Best classical ML (model)	CNN-voting ensemble	*P*-value
Accuracy	0.73 (LightGBM)	0.78	<0.01
AUC	0.79 (CatBoost)	0.85	<0.05
F1 score	0.66 (LightGBM)	0.75	<0.01
Recall	0.64 (LightGBM)	0.72	<0.01

With *p*-values vs. best classical ML.

A similar trend was observed in Dataset 2 (Table 6), where the CNN–Voting Ensemble significantly outperformed the best Classical ML models (CatBoost for Accuracy, AUC, and F1 Score; LightGBM for Recall). Specifically, Accuracy increased from 0.78 to 0.85 (*p* < 0.01), AUC from 0.81 to 0.93 (*p* < 0.01), F1 Score from 0.58 to 0.82 (*p* < 0.01), and Recall from 0.56 to 0.81 (*p* < 0.01). The magnitude of the differences highlights the superiority of the CNN–Voting Ensemble, particularly in F1 Score and Recall, which exhibited the largest relative gains.

**TABLE 6:** Performance results of Dataset 2.

Metric	Best classical ML (model)	CNN-voting ensemble	*P*-value
Accuracy	0.78 (CatBoost)	0.85	<0.01
AUC	0.81 (CatBoost)	0.93	<0.01
F1 score	0.58 (CatBoost)	0.82	<0.01
Recall	0.56 (LightGBM)	0.81	<0.01

With *p*-values vs. best classical ML.

On Dataset 3 (Table 7), the Classical ML models already achieved very strong performance, with Accuracy and AUC exceeding 0.95. Nonetheless, the CNN–Voting Ensemble still yielded modest but statistically significant improvements in most metrics: Accuracy (0.98 vs. 0.96, *p* < 0.05), F1 Score (0.98 vs. 0.94, *p* < 0.05), and Recall (0.99 vs. 0.94, *p* < 0.05). For AUC, the difference between the CNN–Voting Ensemble and Random Forest was negligible (0.98 vs. 0.98, *p* = 0.05), indicating comparable performance on this dataset.

**TABLE 7:** Performance results of Dataset 3.

Metric	Best classical ML (model)	CNN-voting ensemble	*P*-value
Accuracy	0.96 (LightGBM)	0.98	<0.05
AUC	0.98(RandomForest)	0.98	0.05
F1 score	0.94 (LightGBM)	0.98	<0.05
Recall	0.94(Voting Ensemble)	0.99	<0.05

With *p*-values vs. best classical ML.

The statistical tests confirm that the CNN–Voting Ensemble consistently outperformed the strongest baseline Classical ML models across all datasets. The improvements were particularly pronounced in Dataset 1 and Dataset 2, where performance gains were not only numerically larger but also statistically highly significant (*p* < 0.01). In Dataset 3, where baseline models were already near ceiling performance, the ensemble achieved smaller yet significant gains. These results provide strong statistical evidence for the superiority of the proposed CNN–Voting Ensemble.

### 4.4 Model interpretability

To evaluate the contribution of individual features to the diabetes prediction task and better understand their roles in classification, we conducted feature importance analysis. By calculating the weight of each feature within the machine learning models, we were able to identify the most influential variables and assess their impact on the model’s predictive outcomes. Specifically, this study computed feature importance scores and analyzed their behavior across different datasets.

**FIGURE 4:**
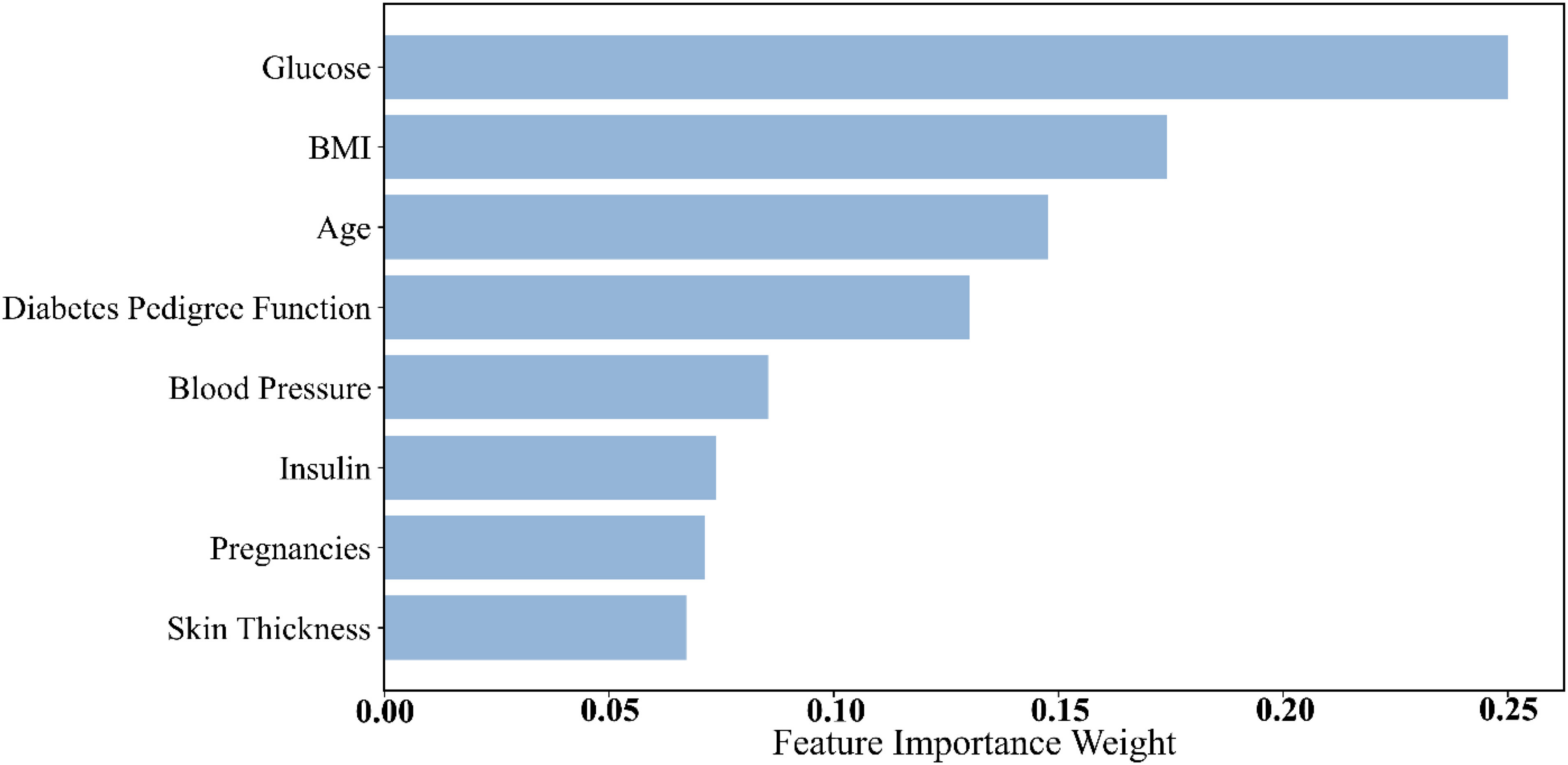
Histogram of feature weights for Dataset 2.

Dataset 2 included several key physiological indicators reflecting an individual’s health status. Feature importance was assessed, and the contribution of each feature to diabetes prediction was quantified. According to the results in Table 8 and Figure 4, glucose (importance score: 0.2500) ranked first, with the highest feature weight. This finding aligns with the physiological foundation of diabetes prediction—glucose level is one of the most critical biomarkers for diabetes. Elevated blood glucose is a hallmark of the condition, making glucose an indispensable contributor to predictive models.

**TABLE 8:** Dataset 2 feature importance weights.

No.	Feature	Importance weights
1	Glucose	0.2500
2	BMI	0.1741
3	Age	0.147
4	Diabetes pedigree function	0.1301
5	Blood pressure	0.0854
6	Insulin	0.0739
7	Pregnancies	0.0714
8	Skin thickness	0.0672

Following glucose, Body Mass Index (BMI) (0.1741) and age (0.147) ranked second and third, respectively. BMI is a key indicator of obesity, which is a major risk factor for diabetes. High BMI is strongly associated with insulin resistance and metabolic syndrome, thus playing an essential role in diabetes risk stratification (45). Age is also a well-established risk factor for diabetes, with incidence increasing significantly after the age of 45 (46). Accordingly, age received a high importance score in the model.

Other features in Dataset 2, such as Diabetes Pedigree Function and blood pressure, also demonstrated strong predictive value. The pedigree function reflects genetic predisposition, a known factor in diabetes development. Blood pressure is closely linked to diabetes complications—hypertension often coexists with diabetes and increases the risk of cardiovascular and renal diseases (47).

**FIGURE 5:**
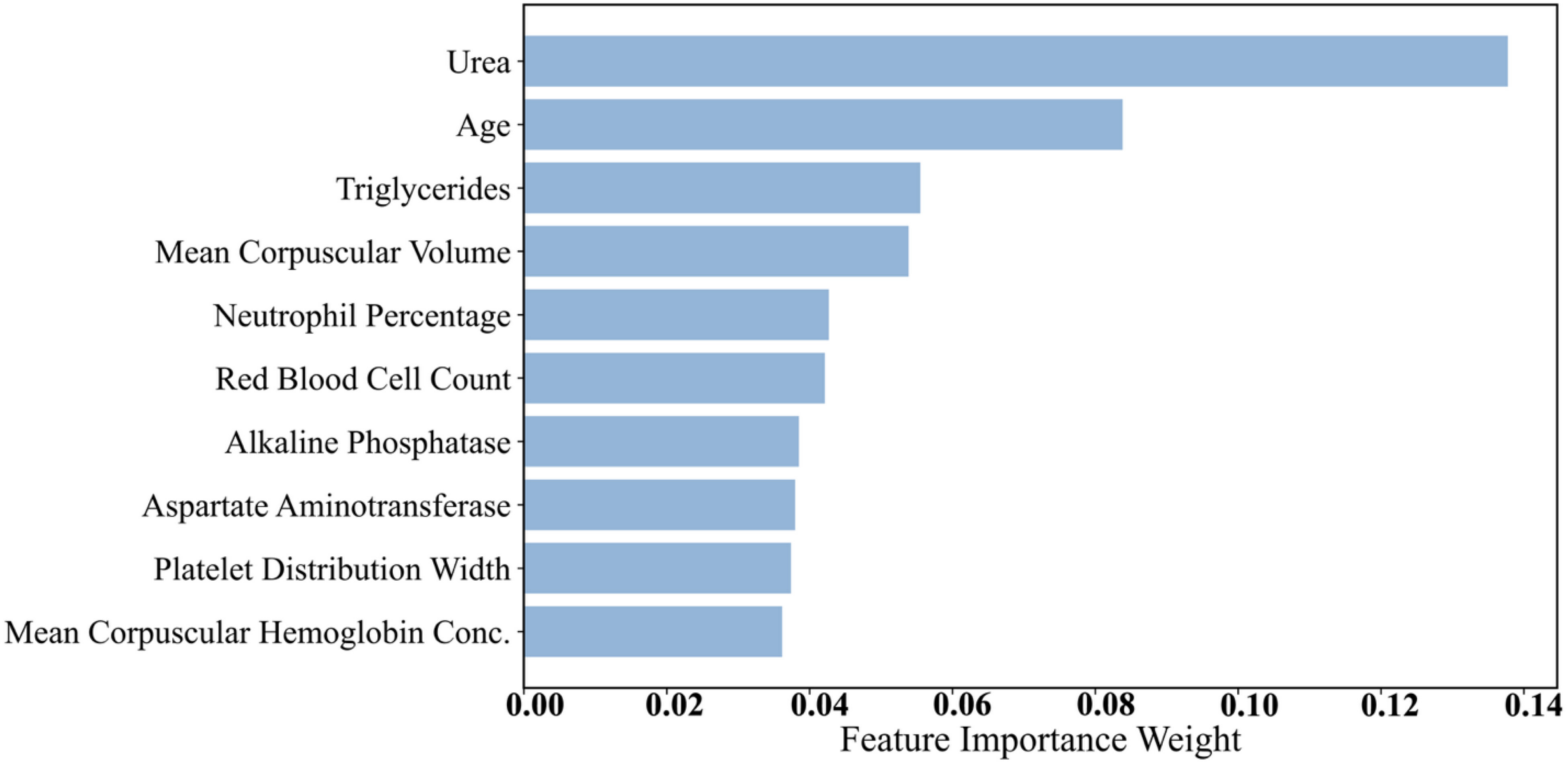
Histogram of feature weights for Dataset 3.

Dataset 3 incorporated a broader range of laboratory test results, increasing feature complexity. As presented in Table 9 and Figure 5, urea (0.1378) emerged as the most important predictor. Urea is a nitrogenous metabolite in the blood, and its concentration level can be a sensitive reflection of the metabolic and excretory function of the kidneys. In several studies, including a comparative study by Kumar et al. (48), it was found that serum urea levels were significantly higher in diabetic patients than in nondiabetic populations, especially in those with a longer duration of disease or poor glycaemic control, and that this elevation was strongly correlated with glycated hemoglobin (HbA1c) levels as well as with the duration of the diabetes (48). This finding suggests that blood urea is not only an early marker for the development of diabetic nephropathy (DN), but also serves as an important bioindicator of the level of diabetes control and risk of complications. Age (0.0838) and triglycerides (0.0555) ranked second and third, respectively. Age remains a strong predictor due to declining insulin sensitivity over time. Triglycerides are an important component of lipid metabolism in humans, and they also play a key role in the development and progression of diabetes. In recent studies, elevated plasma triglyceride levels have been highly correlated with insulin resistance. Under normal physiological conditions, insulin inhibits lipolysis in adipose tissue and reduces the release of free fatty acids (FFAs) (49). However, in insulin resistance, this inhibitory mechanism is impaired, leading to a significant increase in blood FFAs, which further stimulates hepatic synthesis of triglycerides and creates a vicious circle. In addition, abnormally elevated triglycerides can directly exacerbate decreased insulin sensitivity by interfering with insulin signaling pathways (50). This lipotoxic effect is not only a precursor state for the development of type 2 diabetes, but also a risk factor for its chronic complications (e.g., cardiovascular disease, fatty liver, diabetic nephropathy, etc.). In this study, triglycerides were included as a high-impact variable in the mixed model and ranked high in the feature importance analysis, further validating its biological importance in diabetes prediction. Elevated triglyceride levels are often an early indicator of diabetes, making them a valuable feature in predictive modeling.

**TABLE 9:** Dataset 3 feature importance weights.

No.	Feature	Importance weights
1	Urea	0.1378
2	Age	0.0838
3	Triglycerides	0.0555
4	Mean corpuscular volume	0.0538
5	Neutrophil percentage	0.0427
6	Red blood cell count	0.0421
7	Alkaline phosphatase	0.0385
8	Aspartate aminotransferase	0.0380
9	Platelet distribution width	0.0374
10	Mean corpuscular hemoglobin concentration	0.0362
11	Red cell distribution width	0.0317
12	Uric acid	0.0309
13	Albumin	0.0293
14	Lymphocyte percentage	0.0285
15	Hemoglobin	0.0254
16	Globulin	0.0252
17	r-Glutamyl transferase	0.0232
18	Hematocrit	0.0229
19	Monocyte percentage	0.0224
20	Eosinophil percentage	0.0207
21	Creatinine	0.0200
22	Low-density lipoprotein cholesterol	0.0180
23	White blood cell count	0.0174
24	Total cholesterol	0.0167
25	High-density lipoprotein cholesterol	0.0160
26	Basophil percentage	0.0159
27	Alanine aminotransferase	0.0159
28	Plateletcrit	0.0153
29	Mean platelet volume	0.0113
30	Platelet count	0.0110
31	Albumin/globulin ratio	0.0096
32	Male	0.0031
33	Mean corpuscular hemoglobin	0.0019
34	Total protein	0.0018
35	Female	0.0001

Additionally, features such as Mean Corpuscular Volume (MCV) (0.0538) and neutrophil percentage (0.0427) demonstrated notable predictive capacity. MCV reflects red blood cell size, and abnormalities may be associated with chronic conditions, including diabetes. Neutrophil percentage is an immune system marker indicating inflammation, which plays a role in diabetes pathogenesis and complications (51). Recent literature has mentioned that neutrophils can also cause type 1 and type 2 diabetes by promoting chronic inflammation, tissue damage, and immune dysfunction. In type 1 diabetes, neutrophils interact with other immune cells, leading to the destruction of beta cells and the release of pro-inflammatory mediators (52). In type 2 diabetes, persistent hyperglycaemia increases the recruitment and activation of neutrophils, leading to kidney and vascular damage (53, 54). Other features from Dataset 3—such as red blood cell count and platelet distribution width—also ranked relatively high. Platelet-related metrics can reflect coagulation status and vascular health, which are closely related to diabetes complications such as cardiovascular disease and vascular damage.

Overall, glucose consistently exhibited the strongest predictive power across all datasets, which aligns with the physiological mechanisms underlying diabetes. BMI and age also held high importance scores throughout, confirming the central roles of obesity and age in diabetes pathogenesis. Notably, urea stood out in Dataset 3 due to its association with renal function, a critical concern in diabetic complications. Features such as triglycerides, MCV, and neutrophil percentage captured aspects of metabolic status and immune response, further enhancing model performance in complex diagnostic contexts.

Some lower-weighted features in Dataset 3, such as platelet count and white blood cell count, also provided supplementary information for model decision-making. As recent studies have shown, compared with non-diabetic control groups, diabetic patients (including type 1 and type 2) often have higher platelet counts and platelet indices (e.g., mean platelet volume (MPV), platelet distribution width (PDW), platelet-large cell ratio (P-LCR), and plateletcrit (PCT)) (55–57). In individuals with gestational diabetes mellitus (GDM) and type 2 diabetes mellitus (T2DM), persistently elevated white blood cell counts indicate a link between systemic inflammation and the onset of diabetes (58, 59). While their overall importance was lower, they may still contribute to individual predictions, especially in early screening for complications. Variations in these immune and hematological indicators may offer important early clues in diabetes diagnosis.

Through feature importance analysis, we identified the key predictors for diabetes classification. Physiological features such as glucose, BMI, and age consistently provided high predictive value across datasets, confirming their utility in screening and risk assessment. In addition, Dataset 3 features like urea, triglycerides, and MCV further enhanced the model’s capability, especially in predicting diabetes-related complications, thus carrying substantial clinical relevance. These findings provide important interpretability support for the proposed prediction model, helping us understand which features dominate decision-making. By integrating physiological knowledge with feature importance analysis, future diabetes prediction systems can offer more precise early screening and personalized risk evaluation.

## 5 Conclusion

This study proposes an innovative hybrid model that combines convolutional neural networks (CNNs) with ensemble learning methods, significantly improving the accuracy and robustness of diabetes prediction. By combining the deep feature extraction capabilities of CNNs with the advantages of traditional machine learning algorithms, this model performs exceptionally well on three datasets, particularly in handling high-dimensional data and class imbalance issues, demonstrating its strong adaptability and versatility.

Experimental results show that the CNN-Voting Ensemble model outperforms traditional models and standalone ensemble methods across multiple key performance metrics, particularly in terms of accuracy (up to 98%), AUC (0.99), F1 score (0.98), and recall (99%), and achieves the highest MCC across all three datasets (0.56, 0.66, 0.94), reflecting the innovative model’s exceptional predictive capability and efficient diagnostic performance. This indicates that the hybrid model not only improves the performance of diabetes prediction but also provides reliable and interpretable decision support for early diagnosis and risk assessment in real clinical settings.

Additionally, feature importance analysis further validates the critical role of clinically relevant biomarkers (such as blood glucose, BMI, age, and urea) in diabetes prediction, enhancing the model’s clinical interpretability. The model’s high accuracy, stability, and robustness make it a tool with significant clinical application value in diabetes management, particularly when dealing with varying data scales and complexities.

In summary, the hybrid model proposed in this study provides a robust solution for diabetes prediction tasks, significantly outperforming existing methods in terms of accuracy and robustness. Its scalability and interpretability also confer broad clinical application potential, offering new insights for early screening and personalized risk assessment in diabetes management.

## Data Availability

The datasets presented in this study can be found in online repositories. The names of the repository/repositories and accession number(s) can be found below: https://archive.ics.uci.edu/, https://tianchi.aliyun.com/competition/entrance/231638/information.
