# Supplementary material for: Integrating convolutional neural networks with ensemble methods for enhanced diabetes diagnosis: a multi-dataset evaluation
Source: Front Med (Lausanne). 2025 Sep 30;12:1657889. doi: 10.3389/fmed.2025.1657889 (PMC12518227; doi:10.3389/fmed.2025.1657889)
Supplement: Supplementary file 1 [file Data_Sheet_1.pdf]

Appendix A

We also compared performance with the latest modern deep learning architecture, Transformer, which is designed specifically for tabular medical data.

Similarly, in our Transformer architecture, the data preprocessing procedure was consistent with that described earlier. The Adam optimizer was employed to adaptively adjust the learning rate, and the early stopping technique was applied to mitigate overfitting. The rectified linear unit (ReLU) function was used as the activation function.

In contrast to the CNN configuration, the number of attention heads (nhead) in the multi-head attention mechanism varied according to the dataset size, with values of 2, 4, and 8 for Datasets 1, 2, and 3, respectively. Likewise, the embedding dimensions (d_model) were set to 32, 64, and 128 for the three datasets, respectively. The dimensions of the feedforward neural network layers were configured as 128, 256, and 512 for Datasets 1, 2, and 3, respectively.

Table S4. Comparison of Transformer and Fusion Model on Dataset 1

**Category**	**Model**	**Accuracy**	**AUC**	**F1 Score**	**Recall**	**Precision**	**Specificity**	**MCC**	**CI (95%)**
Transformer	Transformer	0.75	0.83	0.66	0.69	0.63	0.78	0.46	[0.68, 0.81]
CNN+Ensemble	CNN-Voting Ensemble	0.75	0.85	0.75	0.72	0.68	0.72	0.56	[0.73, 0.82]

Table S5. Comparison of Transformer and Fusion Model on Dataset 2

**Category**	**Model**	**Accuracy**	**AUC**	**F1 Score**	**Recall**	**Precision**	**Specificity**	**MCC**	**CI (95%)**
Transformer	Transformer	0.95	0.98	0.93	0.94	0.92	0.96	0.89	[0.93, 0.97]
CNN+Ensemble	CNN-Voting Ensemble	0.83	0.93	0.82	0.81	0.96	0.90	0.66	[0.80, 0.88]

Table S6. Comparison of Transformer and Fusion Model on Dataset 3

**Category**	**Model**	**Accuracy**	**AUC**	**F1 Score**	**Recall**	**Precision**	**Specificity**	**MCC**	**CI (95%)**
Transformer	Transformer	0.63	0.7	0.63	0.67	0.6	0.6	0.27	[0.57, 0.69]
CNN+Ensemble	CNN-Voting Ensemble	0.97	0.98	0.98	0.99	0.97	0.99	0.94	[0.97, 0.99]

Across the three datasets, the proposed CNN–Voting Ensemble model consistently demonstrated superior overall performance compared to the Transformer architecture, particularly in scenarios with larger and more complex datasets. While the Transformer achieved strong results on Dataset 2, with notably high accuracy (0.95), AUC (0.98), recall (0.94), and specificity (0.96), its performance dropped substantially on Dataset 1 and Dataset 3. For example, on Dataset 3, the Transformer's accuracy (0.63), AUC (0.70), and MCC (0.27) were markedly lower than those of the fusion model. In contrast, the CNN–Voting Ensemble maintained robust and stable performance across all datasets, achieving particularly outstanding results on Dataset 3, where it reached an accuracy of 0.98, AUC of 0.98, F1 score of 0.98, and MCC of 0.94. These findings suggest that the fusion model is more adaptable and generalizable, especially when handling larger-scale data, and can effectively capture complex feature interactions that the Transformer alone may not fully exploit.
